# Trapped in the extinction vortex? Strong genetic effects in a declining vertebrate population

**DOI:** 10.1186/1471-2148-10-33

**Published:** 2010-02-02

**Authors:** Donald Blomqvist, Angela Pauliny, Mikael Larsson, Lars-Åke Flodin

**Affiliations:** 1 Department of Zoology, University of Gothenburg, Box 463, Gothenburg, 405 30, Sweden; 2 Ljungvägen 3, Väröbacka, 430 22, Sweden; 3 Rannevägen 12, Varberg, 432 95, Sweden

## Abstract

**Background:**

Inbreeding and loss of genetic diversity are expected to increase the extinction risk of small populations, but detailed tests in natural populations are scarce. We combine long-term population and fitness data with those from two types of molecular markers to examine the role of genetic effects in a declining metapopulation of southern dunlins *Calidris alpina schinzii*, an endangered shorebird.

**Results:**

The decline is associated with increased pairings between related individuals, including close inbreeding (as revealed by both field observations of parentage and molecular markers). Furthermore, reduced genetic diversity seems to affect individual fitness at several life stages. Higher genetic similarity between mates correlates negatively with the pair's hatching success. Moreover, offspring produced by related parents are more homozygous and suffer from increased mortality during embryonic development and possibly also after hatching.

**Conclusions:**

Our results demonstrate strong genetic effects in a rapidly declining population, emphasizing the importance of genetic factors for the persistence of small populations.

## Background

Fragmentation of natural habitats is associated with population declines of many species. The resulting small and isolated populations are threatened by extinction for several reasons (reviewed in [1]). Such populations are more vulnerable to demographic and environmental stochasticity. They also face several genetic threats. First, due to restricted mating opportunities, inbreeding becomes more likely. Second, if populations remain small and isolated for many generations, they lose genetic variation necessary to respond to environmental challenges (random fixation or loss of alleles through genetic drift). Third, unfavourable mutations are expected to accumulate because selection operates less efficiently in small populations. Of these processes, inbreeding poses a more immediate threat, whereas genetic drift and mutation accumulation affect the population in the long term [1,2]. Environmental, demographic and genetic factors can interact and reinforce each other in a downward spiral, an extinction vortex [3,4].

Inbreeding has long been suggested to adversely affect naturally outbreeding species [5] and has implications for many aspects of biology, such as plant breeding systems [6] and mating strategies in animals [7-9]. Inbreeding depression refers to the reduction in offspring fitness caused by matings between related individuals and arises from the expression of recessive deleterious alleles in homozygotes or reduced frequency of heterozygote genotypes with superior fitness (e.g. [1,6]). The importance of inbreeding and other genetic mechanisms in population extinction is controversial [1,2]. It has been proposed that species are likely to go extinct for other reasons before deleterious genetic changes will affect them. However, Spielman *et al *[10] reported lower genetic diversity (heterozygosity) in threatened taxa compared to related non-threatened taxa, indicating a link between extinction risk and reduced genetic variation.

There is now compelling evidence from natural populations that inbreeding depression has a marked impact on the performance of individuals, reducing their survival, reproduction and resistance to environmental stress [1,11,12]. Given that inbreeding reduces individual fitness, it may also eventually erode population fitness and increase the risk of extinction. In accordance, reduced population heterozygosity (presumably reflecting higher inbreeding [1,2]) is associated with reduced reproductive fitness of the population [13]. Furthermore, computer projections [14], laboratory experiments with flies and mice, and studies of plants and butterflies in the wild [1,2] suggest that genetic factors influence population extinction risk (but see [15]). However, more detailed work is needed to explore how inbreeding affects the entire life cycle and to test to what extent inbreeding contributes to the extinction vortex of fragmented populations ([1,2], see also [4]).

Here, we analyze the interaction between population decline and genetics in a long-lived vertebrate, the dunlin *Calidris alpina*. Building on long-term population and molecular data from a small metapopulation of the endangered subspecies *C. a. schinzii *(southern dunlin), we examine changes in the genetic constitution during 12 years and their influence on fitness components at different life stages. Our findings demonstrate serious genetic effects in a declining natural population, likely reducing the prospects for its survival.

## Methods

### Study species

The dunlin is a migratory shorebird (suborder Charadrii) with a Holarctic breeding distribution and several recognized subspecies. Dunlins produce clutches of four eggs that hatch synchronously and are cared for by both parents. The precocial and highly mobile chicks usually leave the nest within a few hours after hatching [16]. Average adult life span is 5-7 years (data from populations of *C. a. schinzii *[17,18] and D. Blomqvist, unpublished data), the oldest known bird living almost 20 years [16].

Although the southern dunlin is still common in Iceland and parts of Britain, it has greatly decreased in numbers in the countries surrounding the Baltic Sea [19]. A century ago, dunlins were common in this area, breeding on wet meadows and pastures. Agricultural changes have since resulted in extensive loss of breeding habitat and a subsequent large population decline [19]. The entire Baltic population is estimated at about 1000 pairs (mainly in Denmark, Sweden and Estonia) and is therefore of particular conservation concern [19].

### Study population

We studied a metapopulation of southern dunlins breeding on coastal pastures in SW Sweden (between 58°00'N, 11°34'E and 57°08'N, 12°13'E). We have monitored the size and distribution of local populations since 1989 [20] and began collecting individual-based data on demography, parentage and reproductive success a few years later. Here, we analyze pooled data from all local populations 1993-2004 (Table 1). During this time period, the metapopulation contained a maximum of nine local populations, most of them holding only a few pairs (two sites held 60-88% of all pairs). All identifiable (ringed) immigrants to the local populations were born on the Swedish west coast, i.e. within the metapopulation (D. Blomqvist, unpublished data).

**Table 1:** Population data for southern dunlins and overview of samples used in the genetic analyses

Year	Total no. of pairs*	No. of finger- printed pairs^†^	Total no. of hatchlings	No. of genotyped hatchlings^‡^
1993	35	2	47	1
1994	38	2	54	2
1995	37	0	62	8
1996	24	2	26	2
1997	30	7	42	10
1998	25	2	36	3
1999	32	2	34	3
2000	28	0	34	8
2001	26	6	56	2
2002	26	7	65	13
2003	22	10	42	2
2004	16	0	48	10

*Minimum number based on confirmed breeding attempts (all local populations combined).

^† ^Each pair is only listed the first year it was recorded breeding (most pairs bred in more than one year). Total number of fingerprinted pairs n = 40.

^‡ ^Total number of microsatellite genotyped hatchlings n = 64, representing one randomly selected hatchling of each of 64 parental combinations. All individuals were typed at 9 loci (see Table 2).

### Trapping, ringing and collection of genetic samples

Adults and chicks were trapped, measured and ringed (metal ring plus an individual combination of colour rings) as part of the long-term study. Adults were caught with walk-in traps during incubation; a few were captured together with their chicks after hatching. Chicks were usually caught in or near the nest bowl soon after hatching.

During trapping and ringing in 1997-2003, we also collected samples for genetic analyses. We sampled 20-50 μl blood by puncturing the brachial vein (adults) or the meta-tarsal vein (chicks). The blood was suspended in Queen's lysis buffer [21] and stored at 4°C. Tissue samples, recovered from chicks that died before or during hatching, were kept in absolute ethanol at -20°C until DNA was isolated.

Permissions for trapping and ringing were issued by the Bird Ringing Centre (Swedish Museum of Natural History, Stockholm). Collection of blood samples adhered to the national legal requirements for research with animals (permit numbers: 52/97, 106/99 and M 76-04; Göteborgs and Malmö/Lunds djurförsöksetiska nämnd).

### Reproductive success

Nests were located by observations of incubating birds and by carefully searching suitable areas (see [20]). We estimated hatching dates from observed laying dates or by floating the eggs in water [22], assuming 5 and 22 days for clutch completion and incubation, respectively [16]. We re-visited each nest around the estimated hatching date and recorded the number of hatched eggs, also noting causes of nesting failure, including predation [23], trampling by cattle, flooding or abandonment.

We examined chick survival from hatching to breeding age by analyzing recruitment in a sample of 55 offspring (each genotyped at nine microsatellite loci). Offspring that survived and recruited to the population usually returned within 1-3 years after birth (D. Blomqvist, unpublished data). We assumed that non-returning young died before they reached fledging age or during their first winter(s). This assumption seems reasonable given the strong site fidelity of southern dunlins ([18] and D. Blomqvist, unpublished data). In spite of similar studies of dunlins in southern Sweden [18] and Denmark [24], no more than 280 km away, birds from our study area have never been recorded breeding elsewhere, nor did we find any ringed immigrants from other populations. Furthermore, the annual number of trapped, unringed birds decreased linearly with the total number of previously ringed birds (Spearman rank correlation, r_s _= -0.83, p = 0.0008, n = 12 years), as predicted from capture-recapture models for closed populations [25].

### Pedigrees

Assuming a low frequency of extra-pair fertilizations (as found in most shorebirds [26]), field observations of parentage enabled us to construct social pedigrees of 233 clutches for which both parents had been identified (141 pairs). The parents' pedigrees were checked for common ancestors (i.e. inbreeding). The standard pedigree-based measure of an individual's degree of inbreeding is the coefficient of inbreeding *f*, usually interpreted as the probability of identity by decent of two alleles at a locus (e.g. [27]). Most of our pedigrees were, however, incomplete and too shallow to detect distant inbreeding. Complete information on the pair's parents was available in 23 cases, and in only one case did we know all eight grand-parents. We therefore refrain from using *f *to estimate the individual and population level of inbreeding. Instead, we (1) report the frequency of pairings between first-order relatives (mother-son, father-daughter and brother-sister) as determined by field observations of parentage, and (2) use two types of genetic markers to examine changes in the genetic constitution of the population and relationships between fitness and individual genetic diversity. Some studies have reported that such relationships may be non-linear (e.g. [28]). We therefore also tested several non-linear models (including exponential and quadratic functions), but none of these provided a better fit to our data (results not shown).

### Genetic analyses

We assessed the genetic similarity of mates using band-sharing coefficients derived from multi-locus DNA fingerprints [29], following standard laboratory and scoring procedures (e.g. [8]). Although band-sharing does not give an exact measure of relatedness between two individuals, it provides an index that reflects their relatedness. Such an index, however, still allows statistical testing of e.g. differences in relatedness between groups (e.g. [8,30-34]). Recent studies have often used microsatellite markers to estimate relatedness. However, indices of relatedness based on microsatellite genotyping and DNA fingerprinting frequently correlate, as documented by several previous studies (e.g. [34,35]) and also supported by our findings (see Results).

We hybridized DNA with the multi-locus probe *per *[36] and scored on average 28.5 bands in males (range 10-37) and 29.0 bands in females (range 15-38). Our sample consisted of 40 pairs (Table 1), first formed between 1993 and 2003. We examined the influence of genetics on hatching success in a subsample of 36 pairs. For these pairs, we selected all their first clutches in which at least one egg hatched, thereby removing environmental influences such as predation on hatching rates. We then calculated each pair's total hatching success over the years as: sum of hatchlings/sum of eggs produced. The mean number of analyzed clutches per pair was 1.6 (range 1-6 clutches). In one year, three of the pairs produced a clutch that survived beyond the due hatching date and was subsequently abandoned by the parents (thus resulting in complete hatching failure). These cases may or may not represent inbreeding depression, and we conservatively excluded them from the analysis (including them yielded the same result; not shown).

Individual genetic diversity of offspring was assessed by allelic heterozygosity at microsatellite loci. We genotyped a sample of 76 individuals at nine polymorphic loci (Table 2). Sixty-four of these were chicks used in the subsequent analyses (Table 1 and below), the remaining 12 individuals were only used for assessing the degree of marker polymorphism. Allele frequencies and expected frequency of heterozygotes were calculated using CERVUS v. 2.0 [37]. Two loci deviated from Hardy-Weinberg Equilibrium (homozygote excess; Table 2), possibly indicating non-amplifying alleles (null alleles) or allelic dropouts [38]. We therefore excluded these loci from further analyses (including them yielded qualitatively similar results; data not shown). We used GENEPOP v. 3.1b [39] to investigate potential linkage between loci. After sequential Bonferroni correction [40,41], however, none of the locus pairs were in significant linkage disequilibrium.

**Table 2:** Characteristics of microsatellite loci used to assess individual heterozygosity in southern dunlins (n = 76 individuals)

Locus*	No. of alleles	Allele size range (bp)	**H**_**O**_	**H**_**E**_
Calp2	8	127-147	0.684	0.711
Calp4	5	118-128	0.173^†^	0.451
Calp5	4	112-118	0.500	0.590
Ruff1	9	175-215	0.868	0.842
Ruff6	7	123-147	0.763	0.779
Ruff9	6	180-200	0.763	0.791
Ruff10	6	252-280	0.421^†^	0.649
PGT83^‡^	8	155-171	0.697	0.754
4A11	2	143-145	0.167	0.359

For each locus, the name, the source species from which the locus was originally isolated and its reference are presented. Amplification results are shown as number of alleles, range of allele sizes in base pairs (bp), observed (H_O_) and expected heterozygosity (H_E_).

*Locus name (source species, reference): Calp2-5 (*Calidris alpina*, [54]); Ruff1-10 (*Philomachus pugnax*, [55]); PGT83 (*Calidris canutus*; D.M. Buehler, A.J. Baker, unpublished data; GenBank Accession number AY198173); 4A11 (*Haematopus ostralegus*, [56]).

^†^Significant deviation from Hardy-Weinberg Equilibrium (χ^2 ^= 26.7, df = 1, p < 0.001, and χ^2 ^= 9.5, df = 1, p < 0.01, respectively).

^‡^Primer sequence (5'-3'); F: AATCCGTTTCTGGGGACTGGG, R: TGCCTAATGCTGACTCACACC (A. Pauliny, unpublished data).

**Figure 1:**
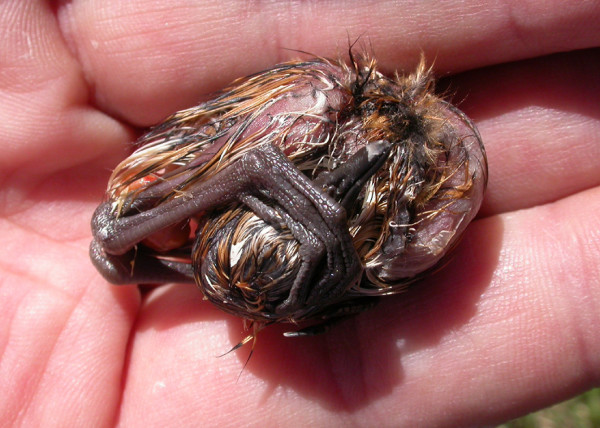
**Dead southern dunlin chick removed from its egg**. Chicks that died during embryonic development were on average more homozygous than those that hatched. Photograph: Angela Pauliny.

Sixty-four of the genotyped individuals each represented one randomly selected offspring from 64 different parental combinations (37 of which were fingerprinted). All of these individuals were typed at all loci, and we therefore calculated multi-locus heterozygosity as the number of heterozygous loci divided by the number of loci examined (i.e. seven). Seven (10.9%) of the genotyped chicks failed to hatch (died before or during the hatching process, Figure 1). Two of the hatched chicks fledged in 2004 and were excluded when analysing recruitment rate, as they might have been alive without us detecting them (our field effort was greatly reduced from 2005 onwards). Of the remaining 55 chicks (born 1993-2003), 26 (47.3%) were recruited to the breeding population.

Microsatellite genotyping was based on PCR reactions carried out in 10 μl 1× *Taq *polymerase buffer B containing 15 ng of template DNA, 0.5 U of *Taq *polymerase (Promega), and final concentrations of the following components: 20 μM (Calp2) or 200 μM (all others) of each dNTP, 1 mM (Calp4) or 1.5 mM (Calp2 and Calp5) or 2 mM (all others) of MgCl_2_, and 0.4 μM (Calp4 and Calp5) or 1 μM (all others) of both forward and reverse primer. One primer of each pair was dye-labelled (WellRED D2-PA, D3-PA or D4-PA; Proligo) and PCR amplification was carried out on an Eppendorf Mastercycler Gradient. All thermal profiles consisted of an initial 2 min denaturation at 94°C and a final 5 min extension step at 72°C, whereas the denaturation (94°C), annealing (varying temperatures, see below) and extension (72°C) steps of each amplification cycle all lasted 30 sec. For all Ruff primers, the following annealing temperatures were used in a touch-down program: 15 cycles at 52-45°C (decreasing with 0.5°C in each subsequent cycle), followed by 25 cycles at 45°C. The PGT83 and 4A11 loci were amplified with 20 cycles at 57-47°C (decreasing with 0.5°C in each subsequent cycle), followed by 20 cycles at 47°C. For the Calp4 and Calp5 microsatellites, the annealing temperature was 61.5°C during 40 cycles (Calp4) and 56°C during 35 cycles (Calp5). The Calp2 locus was amplified at 58°C (5 cycles), followed by 57°C (15 cycles) and finally 56°C (20 cycles). The size of amplification products was determined on a CEQ™8000 Genetic Analysis System (Beckman Coulter) using the Fragment Analysis Module (software version 8.0.52).

## Results

**Figure 2:**
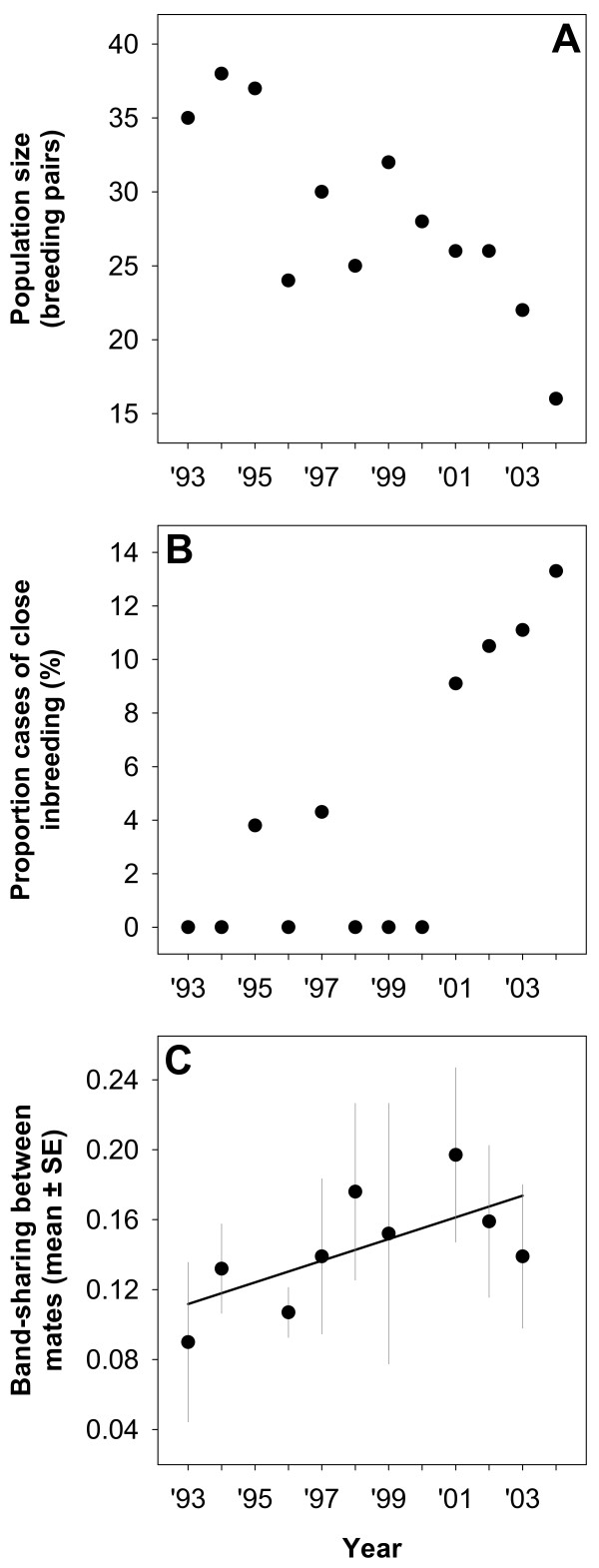
**Population development and genetic similarity of mates 1993-2004 in southern dunlins breeding in SW Sweden**. (A) Total population size (minimum number based on confirmed breeding attempts). (B) Relative frequency of close inbreeding, i.e. matings between first-order relatives (based on field observations of parentage). (C) Yearly mean genetic similarity between mates as assessed by band-sharing values derived from multi-locus DNA fingerprints. Each pair was only included the first year it was recorded breeding (2-10 pairs per year, see Table 1). The regression line is shown for descriptive purposes (slope = 0.66, SE = 0.28).

Our long-term monitoring of the entire population revealed a nearly continuous decline of breeding pairs during the study period (Figure 2A). This decline was associated with increased pairings between related individuals. We recorded matings between first-order relatives in at least six out of 141 pairs (4.3%). The frequency of these incestuous pairings appeared to increase during the study period 1993-2004; four out of six occurred first 2001-2004 (Figure 2B). In addition, two of these four pairs were re-formed on one (2002) and three occasions (2003-2004), respectively. During the last four years (2001-2004), 9.1-13.3% of all pairings represented matings between first-order relatives (Figure 2B).

DNA fingerprinting, including individuals with unknown ancestry, also showed an increase in genetic similarity of mates in the population. Selecting for test only the data from the year when each pair was first formed confirmed an increase in yearly mean genetic similarity during the study period (Figure 2C; Spearman rank correlation, r_s _= 0.69, p = 0.038, n = 9 years). Pairs consisting of first-order relatives, as determined by field observations of parentage, showed on average higher band-sharing values (mean ± SE: 0.40 ± 0.03, n = 4 pairs) than presumably less related pairs (0.12 ± 0.01, n = 36 pairs; Mann-Whitney U test, U = 1, p = 0.001), confirming that band-sharing values from DNA fingerprints can be used as an index of relatedness (see Methods).

**Figure 3:**
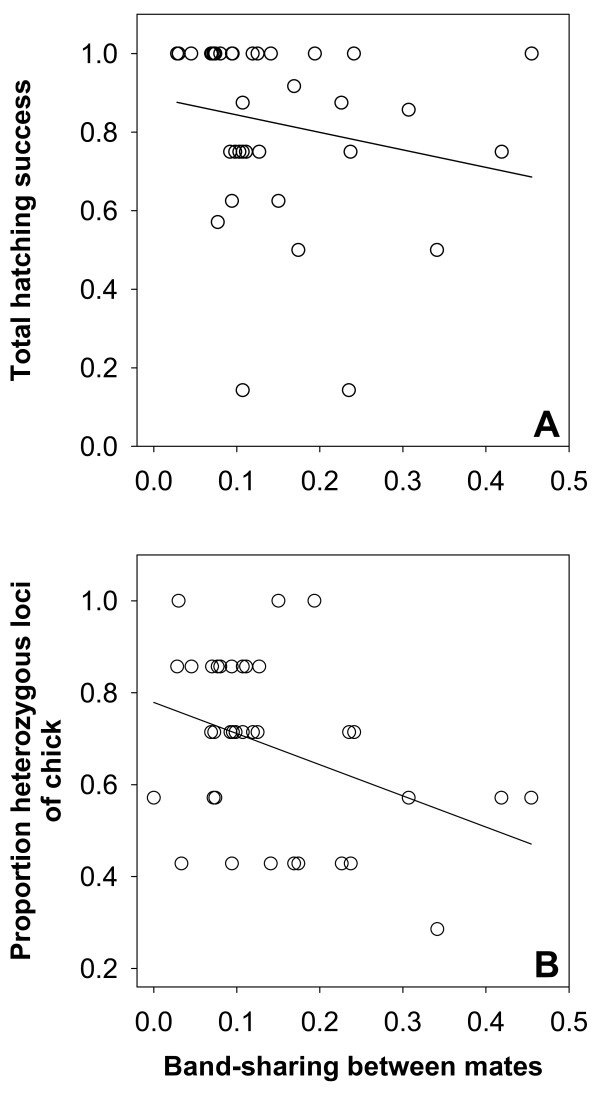
**Hatching success and genetic variation in southern dunlins**. (A) Relationship between total hatching success (sum of hatchlings/sum of eggs, including clutches in which at least one egg hatched) and genetic similarity of mates (band-sharing values derived from multi-locus DNA fingerprints), n = 36 pairs; the regression line is shown for descriptive purposes (slope = -0.20, SE = 0.17). (B) Relationship between allelic heterozygosity of chicks (genotyped at 7 microsatellite loci) and genetic similarity of their parents (DNA fingerprinting), n = 37 chicks from 37 different pairs (one randomly selected chick per pair); the regression line is shown for descriptive purposes (slope = -0.38, SE = 0.16).

**Figure 4:**
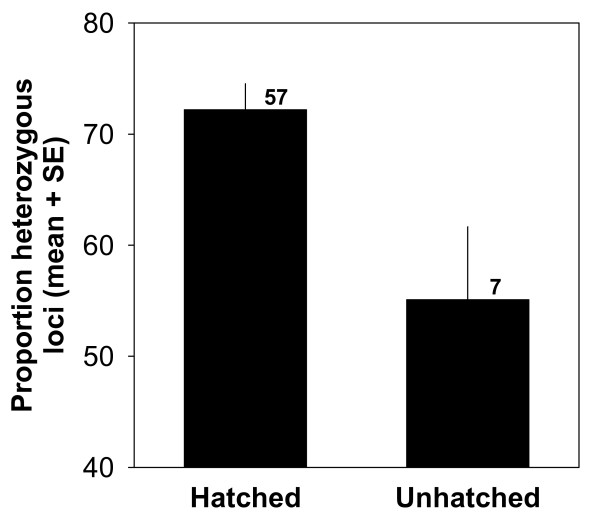
**Individual genetic diversity of southern dunlin chicks that hatched or died during embryonic development (unhatched)**. Genetic diversity was assessed as allelic heterozygosity at 7 microsatellite loci. Numbers above bars denote sample sizes (number of chicks).

We examined the role of genetics on early life stages by first relating hatching success to the genetic similarity of parents. After excluding nests that did not survive until hatching (thus removing environmental influences such as predation on hatching rates), we found that genetically similar pairs suffered reduced hatching success (Figure 3A; Spearman rank correlation, r_s _= -0.38, p = 0.024, n = 36 pairs). Furthermore, genetically similar parents produced offspring with reduced genetic diversity and increased mortality early in life. Thus, genetic similarity of mates (DNA fingerprinting) correlated negatively with the degree of heterozygosity (microsatellites) in their offspring (Figure 3B; Spearman rank correlation, r_s _= -0.33, p = 0.046, n = 37 chicks), and chicks that died during embryonic development showed lower levels of heterozygosity than those that hatched (Figure 4; Mann-Whitney U test, U = 97, p = 0.023, n = 7 and 57 chicks, respectively).

Finally, we assessed the influence of genetics on later life stages by examining the relationship between genetic diversity and survival from hatching to breeding age (recruitment). We found no significant difference in multi-locus heterozygosity between offspring that recruited (mean proportion heterozygous loci ± SE: 0.75 ± 0.03, n = 26) and those that did not (0.70 ± 0.03, n = 29; z = -0.99, p = 0.32). However, previous studies report that heterozygosity at different microsatellite loci may show varying relationships with fitness (e.g. [42]). When we analyzed each marker separately, heterozygosity at one marker (PGT 83) tended to correlate with recruitment. Offspring that were heterozygous at this locus thus returned to the breeding population at a higher rate (56%, 23/41) than those that were homozygous (21%, 3/14; Fisher exact test, p = 0.032), though the difference was not statistically significant after sequential Bonferroni correction [40,41].

## Discussion

In several countries surrounding the Baltic Sea, the loss of dunlin breeding habitat has been halted during recent decades. Pastures and other grassland habitats have even been restored in some areas (e.g. [43-45]). On the Swedish west coast, for example, the available nesting area has remained largely unaltered during the time period examined here (1993-2004, own observations). Thus, the continued decline of this and other dunlin populations in the Baltic Sea region cannot be explained by habitat loss alone. Following the large, initial decrease caused by habitat deterioration [19], it seems likely that genetic and other factors have contributed to a further reduction in population size, making it even more vulnerable to stochastic variation in demography and environmental conditions. The southern dunlin therefore appears to be trapped in an extinction vortex. In an attempt to halt the population decline, we experimentally manipulated nest survival, one of the most important environmental determinants of reproductive success [18,20]. By using protective nest cages in recent years, we were thus able to entirely prevent nest losses due to trampling by cattle as well as significantly reduce the predation rate. Although this resulted in improved nest survival and hatchling production [46], the population has continued to decrease in numbers. Habitat management therefore seems insufficient for preserving this threatened shorebird. Our results indicate that genetic effects are playing a role in the decline of the southern dunlin, even though other factors such as deteriorating wintering areas cannot be ruled out.

Using field observations of parentage and molecular data, we demonstrate an increased frequency of pairings between related individuals during the 12 year study. Such matings, including incestuous inbreeding, resulted in more homozygous offspring with reduced survival during early development (before hatching) and possibly also later in life (recruitment). A recent study of a natural population of great tits *Parus major *confirms that inbreeding can affect the entire life cycle [47]. Although close inbreeding was relatively rare in this population (1.0-2.6% of matings), inbreeding depression was pronounced and translated into reduced hatching success, fledging success, recruitment and production of grand offspring. These and other findings [48] show that studies considering only a part of the life history are likely to underestimate the costs of inbreeding [47]. Our findings seem consistent with theoretical and empirical work predicting that genetic deterioration in small populations influences both individual and population fitness and, thereby, increases their extinction risk [1,2,10,13].

We found that at least 4% of all pairings represented matings between first-order relatives. However, the frequency of close inbreeding does not necessarily provide a good approximation of the population level of inbreeding [49]. Because of the long generation time in dunlins, most of our pedigrees were incomplete and too shallow to detect distant common ancestors. In a population of song sparrows *Melospiza melodia *(a passerine bird with relatively short generation time), pedigrees revealed that 61% of the overall inbreeding was caused by matings among distant relatives [49]. Regardless of the exact level of inbreeding in our study population, field observations and DNA fingerprinting results demonstrated an increased frequency of pairings between related individuals over time. We do not know how accurately genetic similarity between mates reflects overall relatedness in the population. In three other species of shorebirds, fertilizations outside the social pair bond are positively correlated with genetic similarity between mates, suggesting that mate choice aims to avoid inbreeding depression or other negative effects of genetic similarity [8]. If dunlins tend to avoid relatives when choosing a social partner, mean genetic similarity between pair members should underestimate average relatedness in the population.

Inbreeding depression may be most difficult to detect when its effects are greatest. For example, if deleterious genes are expressed very early in development, only less inbred offspring may be left to sample [1]. We circumvented this potential problem by comparing the genotypes of hatched chicks with those that died during embryonic development, finding significantly lower heterozygosity in dead embryos. Together with the negative correlation between offspring heterozygosity and genetic similarity of parents, this result provides a possible mechanism explaining why related parents suffer reduced hatching success, as found here and in several previous studies (e.g. [1]).

Although microsatellites are generally considered selectively neutral (non-coding) markers, this and other studies show that heterozygosity at microsatellite loci may correlate with measures of fitness (reviewed in [50]). Such heterozygosity-fitness correlations may arise in different ways (e.g. [51]), including close chromosomal proximity of the microsatellite locus to a fitness gene (linkage disequilibrium) and non-random associations of genotypes in zygotes (identity disequilibrium). The latter is expected in partially inbred populations, where correlations between heterozygosity and fitness might be equivalent to inbreeding depression in its classical sense [51]. However, recent work questions whether the observed heterozygosity-fitness correlations are caused by inbreeding depression, as invoked by most studies [52,53]. Microsatellite heterozygosity is usually only weakly correlated with pedigree estimates of inbreeding [53], and simulations suggest that such a relationship is only likely to occur in a very restricted parameter space, e.g. when inbreeding events are both frequent and severe [52]. Balloux *et al *[52] proposed that associative dominance through physical linkage with genes under selection is the most important mechanism contributing to heterozygosity-fitness correlations. In dunlins, multi-locus heterozygosity predicted offspring survival until hatching. Interestingly, survival later in life was apparently associated with heterozygosity at only one of the seven loci. The latter finding might support linkage disequilibrium between this marker locus and genes influencing survival after hatching (see e.g. [42,52]), but more work is needed to determine why heterozygosity at neutral markers correlates with fitness in dunlins and other species [42,51-53].

## Conclusions

We have shown that a declining population of a long-lived, endangered vertebrate suffers from substantial negative genetic effects. Our results highlight that ignoring genetics may underestimate the extinction risk of natural populations and thus lead to inappropriate conservation measures [2].

## Authors' contributions

DB conceived and managed the study including data collection, statistical analyses and writing of the manuscript. AP carried out the molecular genetic analyses. AP, ML and LF helped with data collection and writing of the manuscript. All authors read and approved the final manuscript.
